# Rupture of the Left External Iliac Artery and Right Groin Pseudoaneurysm Formation following Angioplasty in a Patient with Neurofibromatosis Type 1 and Undiagnosed Bilateral Phaeochromocytoma

**DOI:** 10.1155/2013/526421

**Published:** 2013-07-25

**Authors:** Brett Doleman, Sushila Kaushal, Asha Patel, James Kirk, John Quarmby

**Affiliations:** ^1^Vascular Surgery, Royal Derby Hospital, Uttoxeter New Road, Derby DE22 3NE, UK; ^2^Vascular Radiologist, Royal Derby Hospital, Uttoxeter New Road, Derby DE22 3NE, UK

## Abstract

Neurofibromatosis type 1 (NF1) is a genetic condition, which affects 1 in every 3000 births. Patients with NF1 are at increased risk of a variety of vascular abnormalities. This report presents the case of a 60-year-old male with NF1 who suffered a left external iliac rupture and a right pseudoaneurysm following angioplasty. In addition, these were further complicated by previously undiagnosed, bilateral phaeochromocytomas. The inherent weakness in vessel wall architecture found in NF1 coupled with the hypertension evident during and after the procedure contributed to haemorrhage and pseudoaneurysm formation. Caution must be taken in such patients when considering vascular intervention.

## 1. Introduction

Neurofibromatosis type 1 (Von Recklinghausen disease) is caused by a mutation in the gene responsible for the production of neurofibromin 1, which is involved in cell signalling. Phenotypic characteristics include formation of neurofibromas, learning difficulties and epilepsy. Neurofibromatosis type 1 (NF1) has an incidence of 1 in 3000 live births, with affected individuals having a 2.7 times increased risk of malignancy compared with the general population  [1].

Although less commonly described, NF1 patients also have an increased risk of vascular lesions, though the actual incidence remains unknown [2]. This increase in vascular lesions results in a higher risk of mortality from vascular disease in younger adults  [3]. The types of vascular lesions are varied and include aneurysms, stenosis, and arteriovenous malformations. Vascular intervention for patients with NF1 is effective and durable over the longer term, although patient survival at 10 years after intervention remains lower than that of the general population  [4].

In addition to an increase in vascular lesions, patients with NF1 have an increased risk of endocrine tumours such as phaeochromocytoma  [5]. Typical symptoms include episodic headaches, sweating, anxiety, and palpitations. Diagnosis is typically made by measuring urinary metanephrines and imaging using computerised tomography (CT) or iodine-131 metaiodobenzylguanidine (I-131 MIBG) scintigraphy.

## 2. Case Presentation

**Figure 1:**
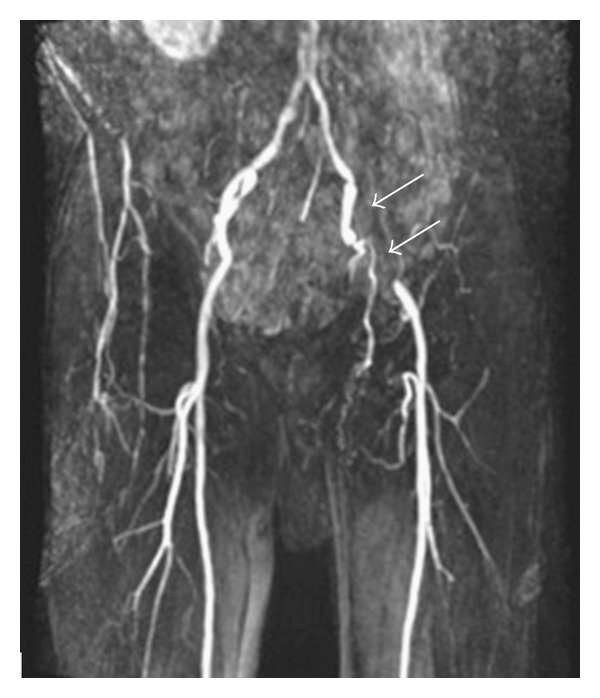
MIP (Maximum Intensity Projection) image from Magnetic Resonance Angiography (MRA) examination demonstrating occlusion of the left external iliac artery (arrows). The left common iliac artery, internal iliac artery, and common femoral artery remain patent.

A 60-year-old male with a background of NF1 (diagnosed around 30 years previously) was referred by his general practitioner with a history of intermittent left lower limb claudication at 100–200 yards. Magnetic resonance angiography (MRA) revealed mild atheromatous disease of both lower limbs, with a 6.5 cm occlusion of the left external iliac artery attributed as being the likely cause of his symptoms (Figure 1). He was admitted for elective angioplasty and stenting of the left external iliac artery. 

Percutaneous access was obtained using ultrasound guidance at the right groin, and pump aortic angiography was performed using a pigtail catheter, confirming the MRA findings. A 7F destination sheath was placed from the right side across the aortic bifurcation, and a 0.018′′ wire was placed within the internal iliac system in order to protect it during the subsequent procedure. The left external iliac artery occlusion was then successfully crossed antegradely using a 4 French Cobra catheter and hydrophilic wire, although this proved difficult due to the firm nature of the occlusion. Predilatation was performed with 3 mm and 5 mm balloons, with no evidence of rupture on subsequent angiography, before placement of a 8 mm × 60 mm self-expanding bare metal stent (ev3 Inc, MN, USA). Postdilatation was performed with a 7 mm balloon.

**Figure 2:**
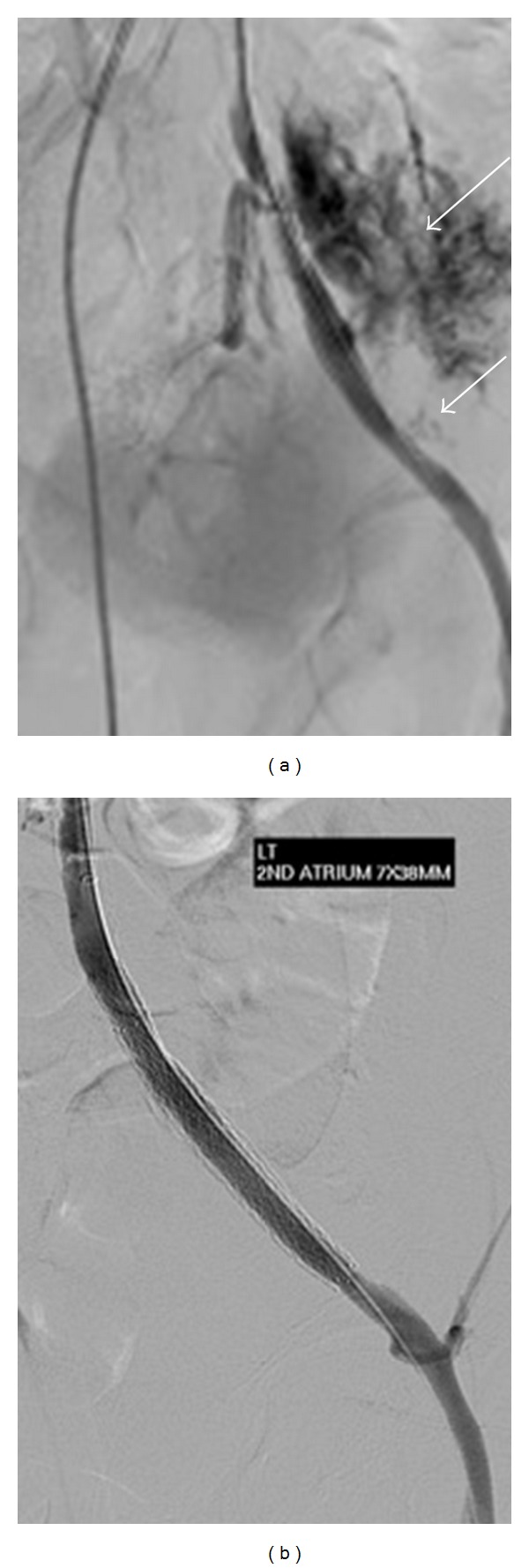
(a) DSA (Digital Subtraction Angiography) image of the left iliac system following insertion of two stents and dilatation. There is vessel rupture with a large amount of contrast extravasation from the proximal portion (upper arrow) of the stented segment and a smaller amount of contrast extravasation from the lower portion (lower arrow). (b) DSA image following insertion of covered stents demonstrating widely patent stented external iliac artery with no further haemorrhage.

 Unfortunately, subsequent angiography demonstrated rupture of the stented segment of the left external iliac artery at two separate sites, with a very large amount of contrast extravasation, indicative of a large retroperitoneal haemorrhage (Figure 2(a)). This was tamponaded immediately with a 7 mm balloon, before deployment of two Advanta V12 7 mm × 38 mm covered stents (Atrium, NH, USA). A good result was achieved with wide patency of the external iliac artery and coverage of the ruptured segments with no evidence of ongoing haemorrhage (Figure 2(b)). The proximal end of the covered stents was deployed across the left internal iliac artery origin. A small nonocclusive filling defect was noted in the tibioperoneal trunk but otherwise patent runoff. A 6F Angioseal VIP device (St Jude Medical, MN, USA) was deployed for haemostasis at the right groin under ultrasound guidance.

The patient was symptomatic with pain and hypotension (BP 70/55 mmHg) at the time of rupture; however, symptoms improved following deployment of the covered stents. Wide bore intravenous access was obtained, and intravenous saline and gelofusine infusions commenced. Analgesia was given in the form of 5 mg of intravenous morphine. He remained stable following the procedure and was transferred to the ward for close observation. Six hours postprocedure, the patient developed left iliac fossa pain and was found to be hypotensive and tachycardic. Fluid resuscitation was commenced and an urgent CT scan was ordered. The precontrast scan confirmed a large retroperitoneal haematoma in the left iliac fossa, extending from the level of the iliac bifurcation to the splenic flexure and measuring approximately 11 cm × 7 cm in the coronal plane. This had areas of high attenuation, suggestive of acute blood but there was no active contrast extravasation or pseudoaneurysm seen either at the stented segment or at the right common femoral artery access site.

The patient remained well overnight; however, the next evening he developed right-sided groin pain and a large pulsatile swelling, which had developed acutely over the course of an hour. An ultrasound scan revealed a bilobed right common femoral artery pseudoaneurysm, approximately 10 cm × 8 cm in diameter. Two hundred units of thrombin (Tisseel, Baxter Healthcare Ltd, UK) were injected, causing thrombosis of the pseudoaneurysm. Repeat ultrasound the next day showed that it had remained thrombosed, with no evidence of persisting leak. A full blood count confirmed that the patient's haemoglobin level was stable and he did not require a blood transfusion.

 At three days postprocedure, the patient experienced some vomiting with an episode of headache and anxiety, which he felt was similar to the symptoms he had had when he suffered a small lacunar infarct the previous year. Neurological examination showed no new focal neurological deficit, and his symptoms were attributed to the stress and anxiety of his current admission. The patient remained well for the rest of his hospital admission and was discharged at four days postangioplasty. At the time of discharge, it was noted that his blood pressure had been particularly labile, even accounting for the events during and postangioplasty. Intraoperatively (ignoring the period of hypotension at the time of stent rupture), his blood pressure varied between 98/60 mmHg and 218/118 mmHg. In the stable postoperative period, it varied between 88/50 mmHg and 174/100 mmHg. In light of the patient's background of NF1, a diagnosis of phaeochromocytoma was considered. He was discharged with a 24-hour urine collection bottle for catecholamine and metanephrine analysis.

**Figure 3:**
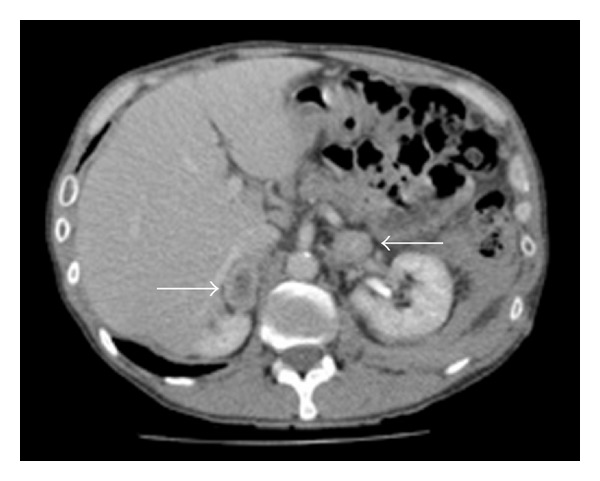
Axial CT image at the level of the adrenal glands demonstrating bilateral adrenal masses (arrows).

 The CT scan taken postangioplasty (Figure 3) for diagnosis of retroperitoneal haemorrhage was re-reviewed at the regional vascular multidisciplinary team meeting. This showed bilateral adrenal masses, measuring 36 × 19 mm axially on the right and 32 × 20 mm axially on the left. The right-sided lesion had a low attenuation centre, whilst the left sided lesion contained foci of high density, suggestive of calcification.

The subsequent 24-hour urinary collection analysis showed significantly raised levels of all catecholamines and metanephrines except the dopamine metabolite 3-methoxytyramine which was within normal range: adrenaline 720 nmol/24 hrs (normal range 0–70 nmol/24 hrs), noradrenaline 1944 nmol/24 hrs (normal range 0–430 nmol/24 hrs), dopamine 3999 nmol/24 hrs (normal range 0–2700 nmol/24 hrs), metadrenaline 5719 nmol/24 hrs (0–1000 nmol/24 hrs), normetadrenaline 8105 nmol/24 hrs (0–3000 nmol/24 hrs), 3-methoxytyramine 1664 nmol/24 hrs (normal range 0–2300 nmol/24 hrs). These results and the findings of the CT scan were discussed with the hospital's biochemistry laboratory; they advised that although bilateral phaeochromocytomas would be rare, in light of this patient's high clinical probability, repeat 24-hour urine collection analysis plus a chromogranin A test should be performed. The levels of three separate chromogranin A assays were mildly raised: 6.9 nmol/L, 6.4 nmol/L, and 6.2 nmol/L (normal range 0–6 nmol/L). Once again, all urinary catecholamines and metanephrines other than 3-methoxytyramine were significantly raised: adrenaline 522 nmol/24 hrs, noradrenaline 1456 nmol/24 hrs, dopamine 3109 nmol/24 hrs, metadrenaline 4547 nmol/24 hrs, normetadrenaline 6355 nmol/24 hrs, and 3-methoxytyramine 1379 nmol/24 hrs. The latter 24-hour urinary collection was performed at exactly 1 month postangioplasty. The fact that the catecholamine and metanephrine levels remained significantly elevated by this point in time would suggest that they were not simply raised as part of the acute stress response of the hospital admission but rather supports the evidence for there being an underlying phaeochromocytoma.

The patient has now been reviewed by a consultant endocrinologist. I-131 MIBG (iodine-131-metaiodobenzylguanidine) scintigraphy demonstrated moderate to high-grade uptake in the enlarged adrenal glands consistent with bilateral phaeochromocytomas. His cutaneous neurofibromas did not exhibit I-131 MIBG uptake; however, low grade uptake was noted in the right paracolic gutter and within the proximal small bowel loops, suggestive of plexiform neurofibromas. He has been commenced on the alpha adrenoreceptor blocker Phenoxybenzamine and is tolerating a dose of 20 mg twice daily with only minor nasal congestion and occasional dizziness. He has been referred to an endocrine surgeon for consideration for bilateral adrenalectomy.

## 3. Discussion

Patients with NF1 are known to be at increased risk of vascular lesions, although actual incidence remains unknown [2]. Death certificate data from the US found that individuals with NF1 aged less than 30 years old had a proportionate mortality ratio of 3.26 for vascular disease compared with the general population [3]. This increased mortality, however, was not evident in the older age groups such as the 60-year-old patient presented here. Despite this, he remained at increased risk of developing a vascular lesion.

 In terms of types and locations of vascular lesions in NF1, of the 31 patients retrospectively reviewed in one clinic, vascular lesions identified included 38 aneurysms, 20 stenoses, 5 arteriovenous malformations, and 5 arteries compressed by tumours. Locations of the lesions included 17 in the aorta, 12 in the renal arteries, 12 in the mesenteric arteries, 10 in carotid-vertebral arteries, 4 intracerebral, and 3 each in the subclavian-axillary and iliofemoral arteries [4]. Our patient presented with left external iliac stenosis, and therefore, as intervention has shown to be effective in the long term in NF1 [4], he was offered treatment with angioplasty in order to improve symptoms of claudication.

The case presented here represents a rare complication of rupture of the left external iliac artery following angioplasty, while using a bare metal stent and balloon. Following this initial rupture, the subsequent development of a right pseudoaneurysm in the context of undiagnosed phaeochromocytoma is described. This patient was at an increased risk of developing a vessel rupture as patients with NF1 have weaker vessel wall architecture. This is thought to be due to the role of smooth muscle and bone marrow cells in neointimal hyperplasia, inflammation, and an exaggerated response to injury causing increased angiogenesis [6]. Indeed, loss of NF1 in mouse models is lethal and thought to involve the upregulation of Ras molecules in the endothelium, which causes uncontrolled cell proliferation [6]. This negatively affects vessel structure and renders them at increased risk of developing a variety of vascular abnormalities.

 In addition to fragile vessel walls, this patient may also have been at increased risk of haemorrhage following such a rupture due to hypertension recorded during the procedure. Hypertension in such patients may be caused by the stress of the procedure itself, essential hypertension, renal artery stenosis, or phaeochromocytoma. Such increases in pressure may contribute to the direct rupture of vessels or, if such ruptures occur, increase the volume of subsequent haemorrhage. Treatment of the rupture presented in this case was achieved using balloon tamponade and the deployment of a two covering stents, which have previously been found to be an effective treatment for such clinical scenarios [7].

One day postprocedure the patient developed a right pseudoaneurysm at the site of the arterial puncture. This again may have been precipitated by the inherent weak vessel wall structure in conjunction with the hypertension both during and after the procedure. Indeed, hypertension has previously been found to increase the relative risk of pseudoaneurysm following femoral artery puncture by 1.52 [8]. As performed during the presentation of this case, the incidence of pseudoaneurysm can be reduced by the use of ultrasound guidance [9] and successful treatment can be achieved with the use of thrombin injection, which is more effective than conventional compression repair [10]. 

 Further complicating this case presentation, was the discovery of previously undiagnosed bilateral phaeochromocytomas. Phaeochromocytoma occurs in 0.1–5.7% of patients with NF1, 9.6% of which have bilateral adrenal tumours [6]. As our patient was experiencing symptoms of headache and anxiety and was found to have episodes of elevated blood pressure, he was investigated for phaeochromocytoma. Subsequent imaging, urinary metanephrines, and plasma chromogranin A confirmed the diagnosis. Extreme hypertension is frequently associated with phaeochromocytoma and was evident in our patient, which as discussed previously, may have contributed to vessel rupture, increased haemorrhage and pseudoaneurysm formation. Indeed, the association of preexisting vessel abnormalities found in NF1 and phaeochromocytoma has previously been reported to cause bleeding from spontaneous vessel rupture [11]. 

With relation to the contrast media used, it has been widely thought that iodinated contrast is potentially hazardous in patients with phaeochromocytomas and that alpha-adrenergic blockade is required prior to contrast administration. However there is little evidence that this is the case with modern contrast agents and it has been demonstrated that nonionic contrast material can be administered safely without prior medication [12].

In conclusion, we present here a case of left external iliac rupture following stent deployment and the development of a right pseudoaneurysm in a patient with neurofibromatosis type 1 with undiagnosed phaeochromocytomas. The underlying increased risk of developing a vascular lesion and inherent frailty of vessel walls coupled with hypertension resulting from the phaeochromocytoma contributed to these described complications. Caution should be taken when undertaking any vascular procedure in patients with NF1. We recommend preoperative screening of hypertensive NF1 patients using urinary catecholamine and metanephrine analysis.
